# Supplementary material for: A Study on the Impermeability of Nanodispersible Modified Bentonite Based on Colloidal Osmotic Pressure Mechanisms and the Adsorption of Harmful Substances
Source: Nanomaterials (Basel). 2023 Jun 11;13(12):1840. doi: 10.3390/nano13121840 (PMC10302080; doi:10.3390/nano13121840)
Supplement: Supplementary file 1 [file nanomaterials-13-01840-s001.zip › nanomaterials-2435850-supplementary.pdf]

A Study on the Impermeability of Nanodispersible Modified Bentonite Based on Colloidal Osmotic Pressure Mecha-nisms and the Adsorption of Harmful Substances

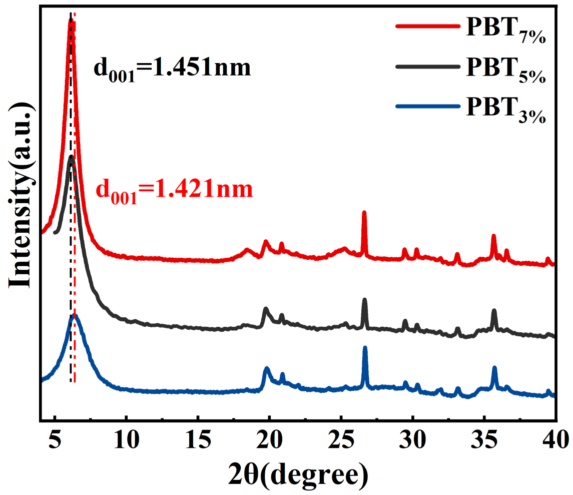

Figure S1. XRD pattern of PBT with different betaine content.

Table S1. Parameters for Langmuir and Freundlich isotherms for the adsorption of phenol on OB, PBT

	K_L	K_f	n
OB	5.71*10^{-4}	0.44352	1.23872
PBT	1.32*10^{-3}	1.31376	1.4842

Table S2.Adsorption kinetics of phenol on OB, PBT.

	q_e(Experiment)	the pseudo-first-order kinetics			the pseudo-second-order kinetics		
		q_e	k_1	R^2	q_e	k_2	R^2
OB	67.38	33.30	0.02469	0.96493	69.97	3.01*10^{-3}	0.9997
PBT	83.33	26.17	0.02568	0.89322	85.61	3.04*10^{-4}	0.9998

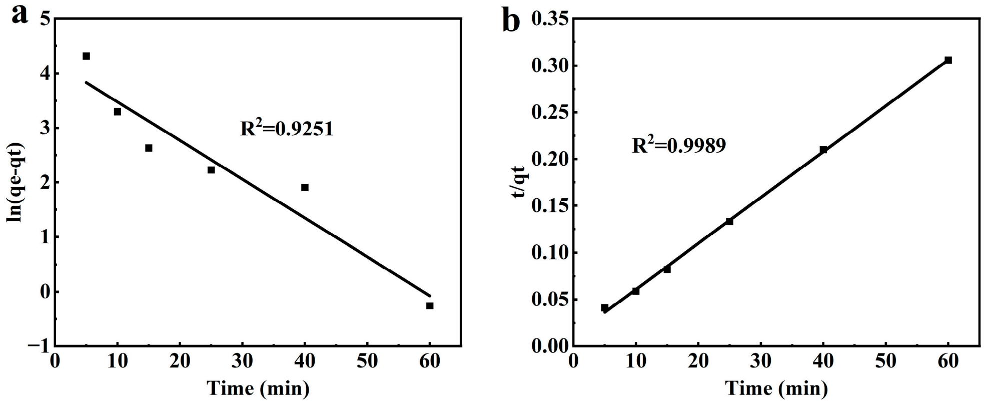

Figure S2. Adsorption kinetics of methylene blue on PBT (a) the pseudo-first-order kinetics

of PBT; (b) the pseudo-second-order kinetics of PBT.

Table S3. Adsorption kinetics of methylene blue on PBT Kinetic parameters.

the pseudo-first-order kinetics		the pseudo-second-order kinetics	
K_1	R^2	K_2	R^2
0.07100	0.9251	0.00526	0.9989

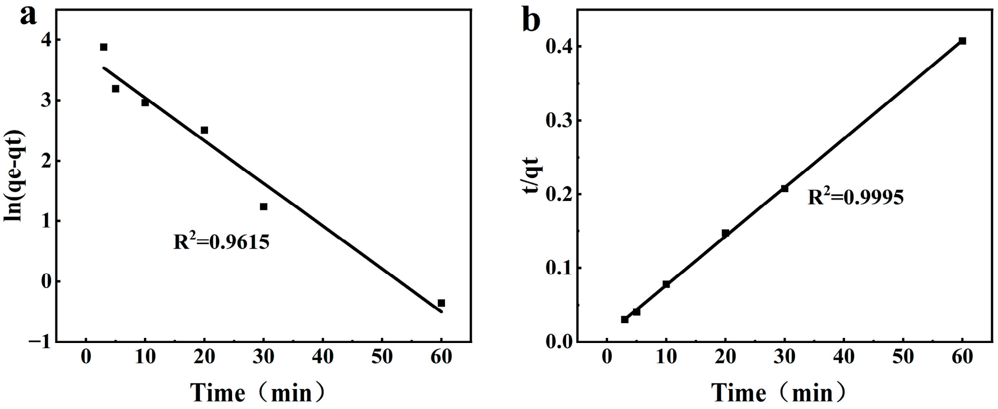

Figure S3. Adsorption kinetics of lead ions on PBT (a) the pseudo-first-order kinetics of PBT; (b) the pseudo-second-order kinetics of PBT.

Table S4. Adsorption kinetics of lead ions on PBT Kinetic parameters.

the pseudo-first-order kinetics		the pseudo-second-order kinetics	
k_1	R^2	k_2	R^2
0.0891	0.9615	0.00438	0.9995
